# Partial loss of Smad signaling during *in vitro* progression of HPV16-immortalized human keratinocytes

**DOI:** 10.1186/1471-2407-13-424

**Published:** 2013-09-18

**Authors:** Diego Altomare, Rupa Velidandla, Lucia Pirisi, Kim E Creek

**Affiliations:** 1 Department of Drug Discovery and Biomedical Sciences, South Carolina College of Pharmacy, University of South Carolina, Columbia, SC, USA; 2 Department of Pathology, Microbiology & Immunology, University of South Carolina School of Medicine, Columbia, SC, USA

**Keywords:** TGF-β signaling, Smads, HPV-mediated transformation, Human keratinocytes

## Abstract

**Background:**

Disruption of the transforming growth factor-beta (TGF-β) signaling pathway is observed in many cancers, including cervical cancer, resulting in TGF-β resistance. While normal human keratinocytes (HKc) and human papillomavirus type 16-immortalized HKc (HKc/HPV16) are sensitive to the growth inhibitory effects of TGF-β, HKc/HPV16 develop resistance to TGF-β1 as they progress *in vitro* to a differentiation resistant phenotype (HKc/DR). The loss of sensitivity to the antiproliferative effects of TGF-β1 in HKc/DR is due, at least partially, to decreased expression of the TGF-β receptor type I. In the present study, we explored in detail whether alterations in Smad protein levels, Smad phosphorylation, or nuclear localization of Smads in response to TGF-β could contribute to the development of TGF-β resistance during *in vitro* progression of HKc/HPV16, and whether TGF-β induction of a Smad-responsive reporter gene was altered in HKc/DR.

**Methods:**

Western blot analysis was used to assess Smad protein levels. In order to study Smad nuclear localization we performed indirect immunofluorescence. In addition, we determined Smad-mediated TGF-β signaling using a luciferase reporter construct.

**Results:**

We did not find a decrease in protein levels of Smad2, Smad3 or Smad4, or an increase in the inhibitory Smad7 that paralleled the loss of sensitivity to the growth inhibitory effects of TGF-β1 observed in HKc/DR. However, we found diminished Smad2 phosphorylation, and delayed nuclear Smad3 localization in response to TGF-β1 in HKc/DR, compared to normal HKc and TGF-β sensitive HKc/HPV16. In addition, we determined that TGF-β1 induction of a Smad responsive promoter is reduced by about 50% in HKc/DR, compared to HKc/HPV16.

**Conclusions:**

These results demonstrate that alterations in Smad protein levels are not associated with the loss of response to the antiproliferative effects of TGF-β in HKc/DR, but that diminished and delayed Smad phosphorylation and nuclear localization, and decreased Smad signaling occur in response to TGF-β in HKc/DR.

## Background

Transforming growth factor beta (TGF-β) is a multifunctional cytokine involved in a variety of cellular processes including cell proliferation, apoptosis, differentiation, epithelial mesenchymal transition, angiogenesis, and metastasis. The overall biological effects of TGF-β are dependent on cell type and context [1-5]. Exposure of most cell types to TGF-β, including epithelial, endothelial, hematopoietic, neuronal cells, and primary mouse embryonic fibroblasts results in inhibition of cell proliferation [6]. TGF-β exerts its effects by binding to receptors on the plasma membrane that belong to the serine/threonine kinase receptor family. Binding of TGF-β to TGF-β receptor type II (TGFBR2) results in the recruitment of type I TGF-β receptors (TGFBR1). This leads to the formation of a membrane complex consisting of the TGF-β ligand dimer, two TGFBR1 and two TGFBR2 receptors. Assembly of this ligand/receptor complex brings the intracellular domains of the receptors in very close proximity, facilitating transphosphorylation and activation of TGFBR1 by the constitutively active TGFBR2 [7]. Activated TGFBR1 then phosphorylates Smad2 and/or Smad3, which are known as receptor regulated Smads. In their phosphorylated state, the receptor-regulated Smads associate with Smad4, a co-Smad. The Smad complex then migrates into the nucleus, where it interacts with a variety of transcription factors, co-activators, co-repressors and chromatin remodeling factors, and binds to Smad-binding elements (SBEs) in the promoter region of target genes, thus regulating the transcription of hundreds of genes [7-11].

Smad7, another protein involved in the TGF-β signaling pathway, is an inhibitory Smad that acts as a negative regulator of the pathway and has been reported to mediate repression of the cytostatic effects of TGF-β [8]. Smad7 expression is induced by TGF-β signaling, thus acting as a negative feedback loop that limits signaling. Smad7 competes with both Smad2 and Smad3 for binding to the activated TGFBR1 and prevents their activation and propagation of the signal into the nucleus [8].

A common characteristic of most human tumors is the loss of sensitivity to the cytostatic effects of TGF-β, which is believed to play an important role in tumor progression and metastasis [12,13]. Alterations in many components of the TGF-β signaling pathway, which lead to TGF-β resistance, have been identified in a variety of human malignancies. Among them are mutations in the genes that encode the TGF-β receptors and Smad proteins, and reduction or loss of TGFBR1, TGFBR2 and Smad expression [12,14]. For example, a loss of Smad4 expression has been reported in cervical cancer tissue [15].

To study the cellular and molecular alterations associated with human papillomavirus type 16 (HPV16)-mediated transformation we utilize an *in vitro* model where normal human keratinocytes (HKc) are immortalized by transfection with HPV16 DNA (HKc/HPV16). HKc/HPV16 progress towards malignancy through several phenotypically defined and reproducible stages that include growth factor independence (HKc/GFI), differentiation resistance (HKc/DR), and ultimately malignant conversion [16-20]. Previous studies in our laboratory demonstrated that HKc/HPV16 are initially as sensitive as normal HKc to the growth inhibitory effects of TGF-β1, but become increasingly resistant during *in vitro* progression [21]. A complete loss of the antiproliferative effects of TGF-β1 is present in HKc/DR, which mimics the TGF-β resistance observed in human cervical carcinoma cell lines [22,23]. In addition, we have previously determined that the loss of growth inhibitory effects of TGF-β1 in HKc/DR is associated with decreased expression of TGFBR1 mRNA and protein, while no change in the expression of TGFBR2 mRNA was found. Importantly, re-expression of TGFBR1 in HKc/DR fully restored growth responses to TGFβ, suggesting that the observed loss of TGFBR1 caused TGFβ resistance in these cells [24,25].

The goal of the present study was to determine whether alterations in protein levels, phosphorylation and nuclear accumulation of Smads could also contribute to the resistance to the antiproliferative effects of TGF-β1 that we observe in HKc/DR. Overall, we found no loss of Smad2, Smad3, Smad4, and no increase in Smad7 during *in vitro* progression of HKc/HPV16. However, we found a delay and a reduction in the phosphorylation of Smad2 after TGF-β1 treatment in HKc/DR, as compared to normal HKc and HKc/HPV16. In addition, we observed a delay in nuclear accumulation of Smad3, and a 50% reduction in the activation of Smad-dependent luciferase expression in HKc/DR following TGF-β1 treatment.

## Methods

### Cell culture and cell lines

Foreskin specimens, derived from elective routine circumcision of neonate boys, were collected in a non-identified fashion from a local hospital. The protocol for foreskin tissue collection and use (PHA #2008-10) was reviewed by the Palmetto Health (Columbia, SC) Institutional Review Board, which determined that this protocol does not constitute human subjects research because it uses non-identified discarded surgical tissue.

Normal HKc were isolated as described previously [26]. Briefly, neonatal foreskins were collected in transport medium: MCDB153-LB medium supplemented with 5% fetal bovine serum (FBS) and 10 μg/ml gentamicin. Connective tissue and fat were removed using a scalpel and the cleaned foreskin was incubated with the epidermis side facing up in 0.25% trypsin (Gibco BRL, Grand Island, NY) diluted 4:1 in complete medium (CM) for 16 to 24 h at 4°C (composition of CM is described below). Epidermis was detached from the underlying dermis using sterile forceps, chopped into small pieces and further disrupted by gentle up and down pipetting in CM. HKc were collected by centrifugation, resuspended into CM and plated in 100-mm tissue culture dishes. Cells were incubated in an atmosphere of 5% carbon dioxide and 95% air at 37°C. Media was changed 24 h post plating and every 48 h thereafter. CM consists of serum-free MCDB153-LB medium supplemented with hydrocortisone (0.2 μM), triiodothyronine (10 nM), transferrin (10 μg/ml), insulin (5 μg/ml), epidermal growth factor (EGF) (5 ng/ml), bovine pituitary extract (BPE) (35–50 μg/ml protein) and gentamicin (50 μg/ml).

Normal HKc were immortalized by transfection with a plasmid containing a dimer of the full-length HPV16 DNA sequence as described in detail previously [16,26]. HPV16 immortalized cells lines (HKc/HPV16) were derived from four different foreskin donors and have been designated HKc/HPV16-d1, -d2, -d4 and -d5 [16,26]. From each of the four HPV16 immortalized lines, growth factor independent cells (HKc/GFI) were selected in CM lacking EGF and BPE, referred to as growth factor depleted medium (GFDM). Furthermore, differentiation resistant cells (HKc/DR) were obtained from HKc/GFI that were selected in CM supplemented with 1.0 mM calcium chloride and 5% FBS [16]. All cell lines were routinely split 1:10 when confluent, medium was changed 24 h after passaging and every 48 h thereafter.

### Preparation of total cellular protein extracts

Cells were grown to about 70% to 90% confluency in 100-mm tissue culture dishes and washed two times with ice-cold phosphate-buffered saline (PBS). Cells were then placed on ice and lysed in 400 μl of RIPA buffer (50 mM Tris–HCl pH 7.5, 150 mM NaCl, 1% NP-40, 0.5% sodium deoxycholate, and 0.1% SDS) that was supplemented with protease inhibitors (complete protease inhibitor cocktail, Roche, Ltd.). Plates were scraped and lysates collected into Eppendorf tubes and mixed for 1 min. After 30 min of incubation on ice, the lysates were mixed again for 1 min and centrifuged at 14,000 × g for 20 min at 4°C. The supernatants were aliquoted and stored in a −80°C freezer until used. Samples used for the assessment of phospho-Smad2 were lysed in RIPA buffer that was supplemented with the protease inhibitor cocktail and with the phosphatase inhibitors sodium fluoride (50 mM) and sodium orthovanadate (1 mM).

### Protein concentration determination in the cellular extracts

Protein concentrations of whole cell lysates were determined using the BCA Protein Assay Kit (Pierce, Part No. 23227) using a microplate format and according to the manufacturer’s recommendations.

### Western blot analysis

Cell lysates were mixed with 5× loading buffer; (Tris–HCl (312.5 mM; pH 6.8), SDS (5%), glycerol (50%), bromophenol blue (0.05%) and beta-mercaptoethanol (β-ME) (25%)). All samples were then adjusted to equal volumes with 1× loading buffer prepared by diluting 5× loading buffer in RIPA buffer. Samples were denatured at 100°C for 5 min and cooled on ice. Samples were run using a 5% stacking gel and resolved on a 10% SDS-polyacrylamide gel. Electrophoresis was performed at 150 V for approximately 1 h using a Mini-PROTEAN II gel apparatus (Bio-Rad Laboratories, Inc) and standard running buffer (25 mM Tris base, 192 mM glycine and 0.1% SDS). The protein standards used for molecular weight assessment were the Precision Plus Protein Standards (Bio-Rad Laboratories). After electrophoresis, the stacking gel was removed, the separating gel was equilibrated for 10 min in refrigerated transfer buffer (25 mM Tris, 192 mM glycine and 20% methanol) and a polyvinylidene fluoride (PVDF) membrane (Bio-Rad Laboratories) was soaked in 100% methanol for 30 sec and equilibrated in refrigerated transfer buffer for 10 min. Proteins were transferred to the PVDF membrane at 80 mA during 16 h using transfer buffer at 4°C. After completion of protein transfer, PVDF membranes were briefly rinsed with 3 changes of PBS containing 0.05% Tween 20 and then blocked at room temperature for 1.5 h using a solution of 5% fat-free milk in the PBS-Tween buffer. For membranes utilized for phospho-Smad2 detection the blocking buffer was supplemented with sodium fluoride (50 mM). Membranes were then incubated overnight at 4°C with primary antibody that was diluted with fresh blocking solution, except for anti-phospho-Smad2 antibody that was added to a solution of 5% bovine serum albumin (BSA) in PBS-Tween. The primary antibodies used were a mouse monoclonal anti-Smad2 diluted 1:2,000 (L16D3; Cell Signaling Technology, Inc.), a rabbit polyclonal anti-Smad3 diluted 1:1,000 (LPC3; Zymed), a mouse monoclonal anti-Smad4 diluted 1:5,000 (B-8; Santa Cruz Biotechnology, Inc.), a rabbit polyclonal anti-Smad7 diluted 1:2,000 (ab5825; Abcam, plc.), a rabbit polyclonal anti-actin diluted 1:20,000 (A2066, Sigma-Aldrich Co. LLC.) and a rabbit monoclonal anti-phospho-Smad2 diluted 1:2,000 (138D4; Cell Signaling Technology, Inc.). The rabbit monoclonal anti-phospho-Smad2 antibody specifically detects endogenous phosphorylated Smad2, with phosphates at serines 465 and 467, which are the phosphorylation target sites of the TGF-β-activated receptor kinase TGFBR1 [27]. Blots probed with anti-actin antibodies were used to confirm equal protein loading. The membranes were then washed at room temperature 5 times with 0.05% Tween 20 in PBS for 5 min per wash and then were incubated at room temperature for approximately 3 h in either an anti-mouse (Vector Laboratories, Inc. - Cat. No. PI-2000) or an anti-rabbit secondary antibody (Vector Laboratories, Inc. - Cat. No. PI-1000). The secondary antibodies were conjugated with horseradish peroxidase (HRP) and were used at a 1:10,000 dilution in 5% fat-free milk/PBS-Tween blocking buffer. A second series of 5 washes at room temperature with 0.05% Tween 20 in PBS for 5 min per wash were followed by chemiluminescence detection using ECL Western Blotting Detection Kit (Amersham Biosciences), according to the manufacturer’s instructions. Subsequently, membranes were placed on X-OMAT AR films (Eastman Kodak) that were developed after exposure. Finally, quantitation of bands was performed by densitometry using ImageJ software [28].

### Preparation of TGF-β1 stocks and TGF-β1 treatment

A stock concentration of 10 μg/ml TGF-β1 was prepared by adding 200 μl of 4 mM HCl solution containing 1 mg/ml BSA to a vial containing 2 μg of lyophilized recombinant human TGF-β1 (R&D Systems, Inc. - Cat. No. 240-B). The TGF-β1 solution was aliquoted and stored at −20°C until use. The TGF-β1 stock solution was freshly diluted 10,000-fold in growth factor depleted medium (GFDM) supplemented only with EGF, but not with BPE (since BPE contains TGF-β) to obtain the 40 pM TGF-β1 concentration utilized in the experiments.

Cells investigated for phospho-Smad2 by Western blotting were grown on 60-mm tissue culture dishes to around 50-60% confluence and then incubated for 24 h in BPE-free CM. Next, cells were treated for 0, 0.5, 1, 2, 4 and 6 h with 40 pM TGF-β1 in BPE-free CM and cell lysates prepared as described above.

### Immunofluorescence and confocal microscopy

Glass coverslips (12 mm) were pre-coated using a 0.01% poly-L-lysine solution (Sigma-Aldrich Co. LLC, product number P4707), according to the manufacturer recommendations, and then placed into a 24-well tissue culture plate where they were soaked overnight in media. The next day normal HKc (10,000 cells), HKc/HPV16 (10,000 cells), or HKc/DR (5,000 cells) were plated on coverslips and allowed to grow until about 50% confluence. Cells were then incubated for 24 h in BPE-free CM and then treated for 0, 5, 15, 30, and 60 min with 40 pM TGF-β1 in BPE-free CM.

Immediately after treatment, cells were rinsed 2 times with ice-cold PBS and fixed for 30 min on ice with 4% neutral paraformaldehyde in PBS. After fixation, cells were washed with PBS (3 × 5 min) and then permeabilized with a solution of Triton X-100 (0.1%) and glycine (0.05 M) in PBS (2 × 10 min). Cells were washed with PBS (2 × 2 min) and subsequently blocked for 45 min with normal goat serum (5%) and BSA (1%) diluted in PBS (blocking solution). A mouse monoclonal anti-Smad4 antibody (B-8; Santa Cruz Biotechnology, Inc.) was diluted 1:150 in 5-fold PBS-diluted blocking solution, added to the cells, and then incubated overnight at 4°C. The following day, cells were washed with PBS (3 × 5 min) and incubated at room temperature for 1 h in a secondary antibody solution, which was prepared by diluting an Alexa Fluor 488 (AF488) conjugated anti-mouse antibody 1:250 in 5-fold PBS diluted blocking solution. Cells were rinsed with PBS (2 × 5 min) and then subjected to a second round of staining for Smad3 using the same conditions. The primary antibody used was a rabbit polyclonal anti-Smad3 diluted 1:200 (LPC3; Zymed) and the secondary antibody was an anti-rabbit conjugated with cyanine 3 (Cy3) diluted 1:250. After incubation with the secondary antibody, cells were rinsed with PBS (2 × 5 min); DNA was stained with DAPI (Molecular Probes) for 15 min and rinsed again with PBS (3 × 5 min). Finally, coverslips were mounted on glass slides using a DABCO containing mounting media. The edges of the coverslips were sealed with nail-polish and allowed to air dry. Cells were imaged on a LSM Meta 510 confocal microscope (Carl Zeiss, Inc.).

Nuclear Smad3 and nuclear Smad4 were later quantified in the acquired confocal pictures using ImageJ software [28]. First, nuclear areas were determined using threshold gating in the DAPI channel. The resulting nuclear areas were then copied to both the Cy3 (Smad3) and AF-488 (Smad4) channels and fluorescence intensities, as well as corresponding areas, were quantified. Intensities for each nucleus were then corrected to its corresponding area. An average of 71 nuclei (range 56–89) was quantified per time point per cell line. Finally, absolute levels were converted to relative values within each time course, having as reference (100%) the maximum level in the time course.

### Transient transfection and luciferase assays

All four HKc/HPV16 and their corresponding HKc/DR lines were plated in 6 well plates and transiently transfected using TransFast (Promega, Madison, WI) in triplicate wells per experimental condition with p6SBE-Luc or P6SME-Luc reporter constructs, along with pRL-SV40 Renilla luciferase (Promega). The p6SBE-Luc and p6SME-Luc constructs, which contain six intact (p6SBE) or mutated (p6SME) Smad-binding-elements (SBE) cloned into the pGL3 plasmid (Promega), were a gift of Dr. Scott Kern. The next day, cells were treated without or with 40 pM TGF-β1 (R&D Systems). Cells were harvested after 22 h of treatment and the lysate assayed for luciferase activity using the Dual Luciferase Assay System (Promega) according to manufacturer’s instructions. The Firefly luciferase values, measured in Relative Light Units (RLU), were normalized against Renilla luciferase activity to control for transfection efficiency.

## Results

### Protein levels of Smad2, Smad3, Smad4 and Smad7 are comparable among normal foreskin keratinocytes established from different donors

We performed Western blot analysis in order to determine whether or not basal protein levels of Smad2, Smad3, Smad4 and Smad7 were comparable among foreskin keratinocytes established from different donors. Protein levels of Smad2, Smad3, Smad4, and Smad7 were comparable among the eight individuals studied (data not shown).

### Protein levels of Smad2, Smad3, Smad4 and Smad7 are not dramatically changed during *in vitro* progression of HPV16-immortalized human keratinocytes

**Figure 1:**
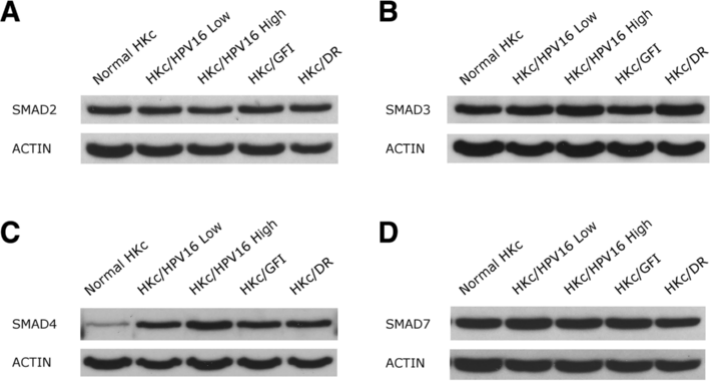
**Western blot analysis of Smads during *in vitro *progression of HKc/HPV16.** Smad protein levels in whole-cell extracts prepared from normal HKc, low passage (less than 40) and high passage (greater than 100) HKc/HPV16, HKc/GFI, and HKc/DR were determined by Western blotting. Total protein (30 μg per well) was loaded and resolved in a 10% polyacrylamide gel. After transferring proteins to PVDF membranes, the Smads were detected using anti-Smad antibodies: **A** – Smad2, **B** – Smad3, **C** – Smad4, and **D** – Smad7. Actin was used as a loading control.

We have previously reported that HKc/HPV16 progressively become resistant to the antiproliferative effects of TGF-β1 during *in vitro* progression through HKc/GFI and HKc/DR stages [21,24]. To determine if altered protein levels of Smad2, Smad3, Smad4 and Smad7 may be a factor leading to TGF-β resistance, we studied the steady state levels of the Smads by Western blot analysis of whole cell lysates. We assessed Smad levels in low and high passage HKc/HPV16, HKc/GFI, and HKc/DR, in four independently established HKc/HPV16 lines originating from different keratinocyte donors [16] in comparison with normal HKc. Representative results are shown in Figure 1. Smad2 and Smad3 protein levels were found not to change during progression of HKc/HPV16 (Figure 1A, 1B). We observed a consistent increase of Smad4 protein expression in HKc/HPV16, HKc/GFI, and HKc/DR compared to normal HKc (Figure 1C). Finally, we found similar levels of Smad7 protein in HKc/HPV16 and HKc/GFI compared to normal HKc, with levels of Smad7 protein decreasing slightly in HKc/DR (Figure 1D).

### Nuclear trafficking of Smad3 and Smad4 after TGF-β1 treatment of normal HKc, HKc/HPV16 and HKc/DR

**Figure 2:**
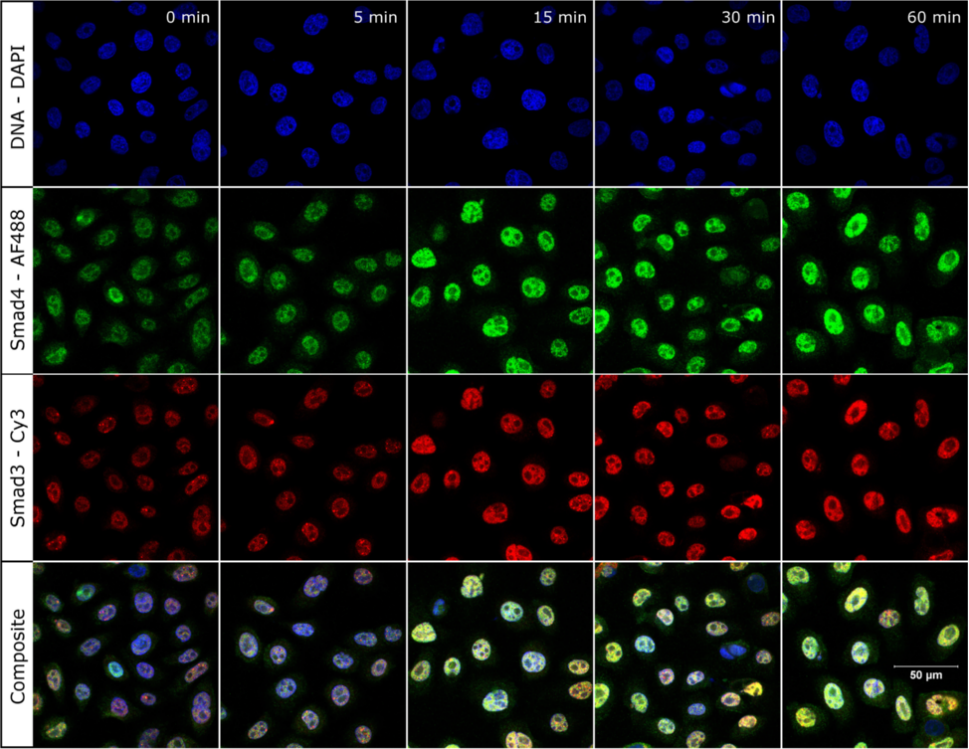
**Nuclear accumulation of Smad3 and Smad4 in HKc/HPV16 after treatment with TGF-β.** HKc/HPV16 were immunostained for Smad3 (Cy3, red) and for Smad4 (Alexa Fluor 488, green) at various times (0 to 60 min) after treatment with 40 pM TGF-β. Nuclei were visualized with DAPI (blue).

**Figure 3:**
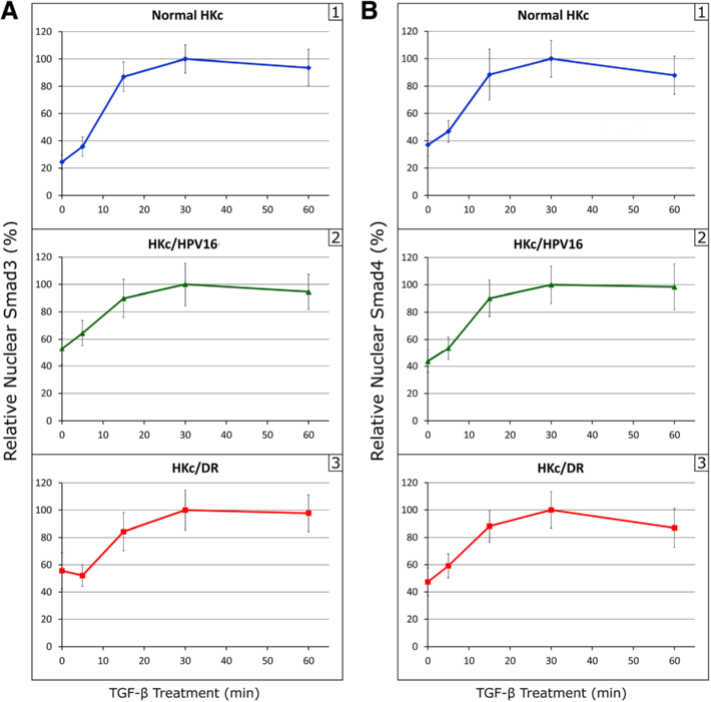
**Nuclear accumulation of Smad3 and Smad4 in normal HKc, HKc/HPV16 and HKc/DR following treatment with TGF-β.** After treatment with 40 pM TGF-β for various times (0 to 60 min), cells were immunostained for Smad3 **(A)** and Smad4 **(B)**, and nuclei were visualized with DAPI. Nuclear accumulation of Smad3 and Smad4 was determined using ImageJ software. 1 Normal HKc; 2 HKc/HPV16; 3 HKc/DR.

We next used indirect immunofluorescence microscopy to compare the nuclear accumulation of Smad3 and Smad4 in normal HKc, HKc/HPV16 and HKc/DR at various times (0 to 60 min) following TGF-β1 treatment. A representative example of the time course of the nuclear accumulation of Smad3 and Smad4 following TGF-β1 treatment is shown in Figure 2 for HKc/HPV16. Nuclear accumulation of Smad3 and Smad4 is evident as early as 5 min of TGF-β1 treatment, with marked nuclear accumulation by 15 min, and sustained nuclear localization up to 60 min (Figure 2). To quantify nuclear Smad3 and Smad4 accumulation over time in normal HKc and all four HPV16 immortalized lines, we used ImageJ software to analyze the immunofluorescence images. For comparison and normalization purposes, we set to 100% the maximum nuclear fluorescence signal obtained for Smad3 or Smad4 during the time course experiment. The intensities observed at the other time points were then expressed relative to the maximal intensity in each time course. A representative time course is shown in Figure 3: Smad3 began to accumulate into the nucleus as early as 5 min after the start of TGF-β1 treatment in normal HKc (Figure 3A, panel 1) and HKc/HPV16 (Figure 3A, panel 2). Maximal nuclear Smad3 accumulation was observed in normal HKc and HKc/HPV16 after 30 min of TGF-β1 treatment (Figure 3A, panels 1 and 2). In contrast, nuclear accumulation of Smad3 in HKc/DR was slightly delayed. Nuclear Smad3 levels remained unchanged in HKc/DR following 5 min of TGF-β1 treatment, although maximal nuclear accumulation was still observed at 30 min (Figure 3A, panel 3).

The time course of Smad4 nuclear accumulation was similar among normal HKc, HKc/HPV16, and HKc/DR, with maximal nuclear accumulation of Smad4 occurring following 30 min of TGF-β1 treatment (Figure 3B, panels 1–3).

### Maximal Smad2 phosphorylation after TGF-β1 treatment is delayed in HKc/DR as compared to normal HKc and HKc/HPV16

**Figure 4:**
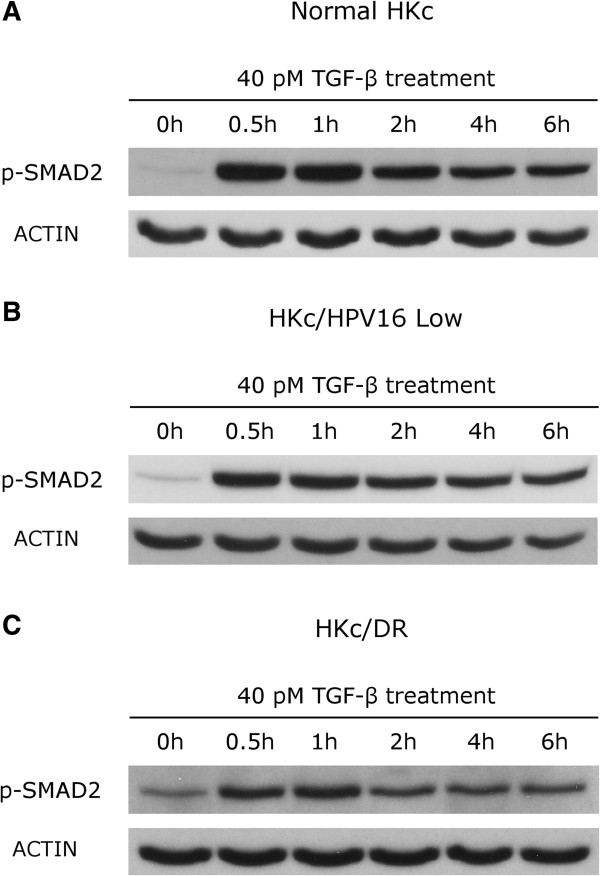
**Time course of Smad2 phosphorylation after TGF-β treatment of normal HKc, low-passage HKc/HPV16 and HKc/DR.** Normal HKc, low-passage (< 40) HKc/HPV16 and HKc/DR were treated with 40 pM TGF-β for the times indicated. Whole-cell lysates were analyzed for phospho-Smad2 by Western blot analysis. Total protein (25 μg/well) was loaded and resolved in a 10% polyacrylamide gel. After transfer to a PVDF membrane, phospho-Smad2 was detected with a rabbit anti-human phospho-Smad2 antibody. Representative time courses are shown for normal HKc **(**panel **A)**, low-passage (<40) HKc/HPV16 **(**panel **B)** and HKc/DR **(**panel **C)**. Actin was used as a loading control.

We also investigated the kinetics of Smad2 phosphorylation after treatment with TGF-β1 in normal HKc, HKc/HPV16, and HKc/DR. Smad2 phosphorylation was assessed by Western blots of whole cell lysates from cells treated for various times (0 to 6 h) with TGF-β1. The maximum level of Smad2 phosphorylation in normal HKc and HKc/HPV16 was observed after 30 min of TGF-β1 treatment and began to decline by 60 min (Figure 4, panels A and B). In contrast, at 30 min HKc/DR had reached only 87% of maximal Smad2 phosphorylation and the peak of Smad2 phosphorylation did not occur until 1 h of TGF-β1 treatment (Figure 4, panel C). Densitometry analysis of multiple Western blots showed that these results were reproducible across six normal HKc strains examined, and four HKc/HPV16 lines with their corresponding HKc/DR lines. These results demonstrate that TGF-β1 signaling is somewhat delayed in HKc/DR compared to normal HKc and HKc/HPV16.

### Phosphorylation levels of Smad2 after TGF-β1 treatment are reduced in HKc/DR as compared to normal HKc and HKc/HPV16

**Figure 5:**
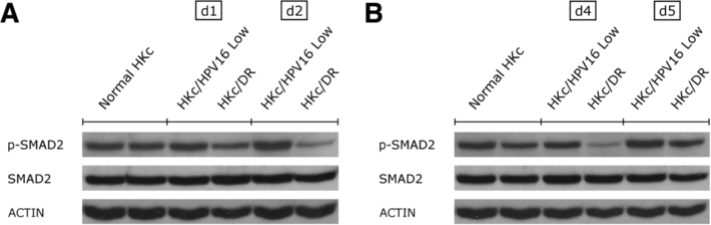
**Western blot analysis of Smad2 phosphorylation in normal HKc, HKc/HPV16, and HKc/DR after 6 h of TGF-β treatment.** Normal HKc, low-passage (< 40) HKc/HPV16, and HKc/DR were treated for 6 h with 40 pM TGF-β. Whole-cell extracts were collected and total protein (25 μg/well) was resolved in a 10% polyacrylamide gel. A rabbit anti-phospho-Smad2 antibody, a mouse anti-Smad2 monoclonal antibody, and a rabbit anti-actin polyclonal antibody were utilized to detect phospho-Smad2, total Smad2, and actin, respectively. Panel **A** shows two normal HKc strains, and HKc/HPV16 and HKc/DR from two donors (d1 and d2). Panel **B** shows the results from two additional normal HKc strains, as well as HKc/HPV16 and HKc/DR from two additional donors (d4 and d5).

We compared the extent of Smad2 phosphorylation to total Smad2 protein in seven normal HKc strains derived from different donors, and four HKc/HPV16 lines with their corresponding HKc/DR following treatment with 40 pM TGF-β1 for 6 h. We observed comparable levels of Smad2 phosphorylation among the normal HKc strains, four of which are shown in Figure 5. Also, comparable levels of phospho-Smad2 between normal HKc and HKc/HPV16 were observed (Figure 5, panels A and B). In contrast, Smad2 phosphorylation was reduced in HKc/DR as compared to normal HKc and HKc/HPV16 (Figure 5, panels A and B). The levels of total Smad2 protein expressed after 6 h of TGF-β1 treatment were comparable among normal HKc, HKc/HPV16 and HKc/DR (Figure 5, panels A and B).

### TGF-β1 induction of a Smad-responsive luciferase reporter construct in HKc/DR is reduced by approximately 50% in comparison with HKc/HPV16

**Figure 6:**
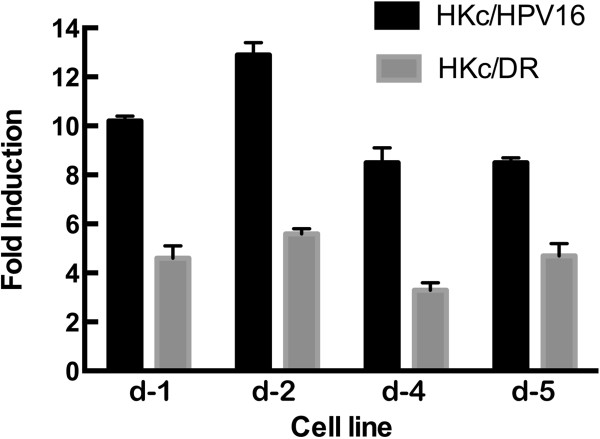
**TGF-β activation of a Smad-responsive luciferase reporter construct in HKc/HPV16 and HKc/DR.** Four HKc/HPV16 and their corresponding HKc/DR lines (d-1, d-2, d-4 and d-5) were transiently transfected in triplicate wells per experimental condition with the p6SBE-Luc reporter construct and pRL-SV40. Cells were allowed to grow in CM for 24 h after transfections. Following this, they were treated without or with 40 pM TGF-B1 for 22 h and harvested for luciferase activity. Firefly luciferase values, measured in Relative Light Units (RLU), were normalized against Renilla luciferase values, and the results expressed as fold induction over control.

Finally, we explored the ability of TGF-β1 to induce the activity of a Firefly luciferase gene under the control of the 6SBE element (p6SBE-Luc). As a control, we used a plasmid structurally identical to the p6SBE-Luc, but in which all six SBEs had been mutated (p6SME-Luc). No induction of luciferase activity was detected in cells transfected with p6SME-Luc and treated with TGF-β1 (data not shown). Induction of luciferase activity was observed in all HKc/HPV16 and HKc/DR lines treated with TGF-β1 (Figure 6). However, while luciferase induction was 8- to 12-fold in HKc/HPV16, it was only 3.3- to 5.6-fold in HKc/DR.

## Discussion

Our laboratory has developed an *in vitro* model of HPV16-mediated human cell transformation in which normal HKc transfected with HPV16 DNA, HKc/HPV16, progress towards malignancy through HKc/GFI and HKc/DR stages [16-18]. HKc/HPV16 are initially as sensitive to the cytostatic effects of TGF-β1 as normal HKc but become increasingly resistant to the antiproliferative effects of TGF-β1 during *in vitro* progression [21]. We have previously linked resistance to growth control by TGF-β1 at the HKc/DR stage to reduced mRNA expression of TGFBR1 but not TGFBR2 [24]. Reduced mRNA expression of TGFBR1 in HKc/DR is not the result of mutations in or hypermethylation of the TGFBR1 promoter, or of changes in the protein levels of the transcription factors Sp1 or Sp3, which drive TGFBR1 expression [25]. The Smads are the intracellular mediators of TGF-β signaling [7,8,10,11]. The goal of the present study was to explore whether alterations in Smad protein levels, as HKc/HPV16 progress through the HKc/GFI and HKc/DR stages, may contribute to the loss of sensitivity to the growth inhibitory effects of TGF-β. In addition, we studied nuclear trafficking of Smad3 and Smad4 in HKc/HPV16 and HKc/DR as well as the kinetics of Smad2 phosphorylation in these cells following TGF-β1 treatment.

Smad2 mRNA expression has been found reduced in 22% of cervical carcinomas, as compared to normal cervix [29], while another study reported “weak” Smad2 protein levels in 33% of cervical tumors [30]. However, no association between Smad2 protein expression in cervical tumors and clinicopathological characteristics such as lymph node status, tumor size, disease recurrence, degree of infiltration and HPV type was found [30]. In our *in vitro* model system we observed no significant reduction of Smad2 protein expression as the cells progress through the HKc/GFI and HKc/DR stages. Thus, we conclude that a reduction in Smad2 does not contribute to the progressive loss of sensitivity to the antiproliferative effects of TGF-β1 that we observe as HKc/HPV16 progress *in vitro*[21]. These findings could suggest that decreased protein levels of Smad2 found in cervical carcinomas is a late event in HPV-mediated disease [30]. This view is supported by our finding that, although differentiation resistant, HKc/DR are not tumorigenic [19,20].

Mutation of the Smad3 gene is very rarely found in human cancer [31]. However, the finding that Smad3 protein is absent in T-cell acute lymphoblastic leukemia (T-ALL), which results in an impaired inhibitory effect of TGF-β on T-cell proliferation, supports the notion of a tumor-suppressing role of Smad3 in at least this disease. Interestingly, the loss of Smad3 in T-ALL is not caused by either mutation or a decrease in its mRNA expression [32]. More evidence supporting the tumor-suppressing role of Smad3 comes from experiments with Smad3-deficient mice, where Smad3 deficiency alone is not enough to initiate tumorigenesis, but decreased Smad3 expression augmented the risk of tumorigenesis when associated with alterations in other genes involved in cellular proliferation and apoptosis [33,34]. In addition to its inhibitory role on cell proliferation, Smad3 can exert a tumor suppression function in hepatic cells by downregulating the antiapoptotic protein BCL2, which results in TGF-β-mediated apoptosis [35]. In our model of HPV16-mediated transformation, we did not find a consistent reduction of Smad3 protein levels as the cells progress *in vitro*. Therefore, alterations of Smad3 protein levels are not likely involved in the progressive reduction of the growth inhibitory response to TGF-β that takes place in this model.

Another protein involved in the transmission of TGF-β signaling from the plasma membrane to nucleus is Smad4 [7-11]. Studies have found homozygous deletions of Smad4 in 30% of pancreatic tumors, and inactivating intragenic mutation in conjunction with loss of the other allele in another 20% of cases [36]. Mutations of the Smad4 gene in other tumor types are less frequent, with a 16% rate in biliary tract cancers, 13% in colorectal carcinomas, 12% in breast cancers, bladder cancers and ovarian cancers, 7% in lung cancers, 6% in hepatocellular carcinoma and 4% in cervical cancers [14]. Studies in cervical tissue have shown similar levels of Smad4 mRNA expression in non-malignant and premalignant tissue. However, Smad4 expression is decreased or lost in 90% of cervical squamous cell carcinomas [15]. An immunohistological study in cervical squamous cell carcinomas revealed a significant correlation of weak cytoplasmic Smad4 staining with both the presence of positive lymph nodes and recurrent disease [30]. Furthermore, absence of nuclear Smad4 protein expression strongly correlated with tumors size and infiltration depth [30]. Both weak cytoplasmic Smad4 and the absence of nuclear Smad4 staining were associated with poor survival in cervical cancer patients [30]. The HPV16-positive SiHa human cervical carcinoma cell line is refractory to growth inhibition by TGF-β, which is explained, at least in part, by reduced expression of Smad4 in these cells [37]. Transfection of SiHa with a Smad4 expression construct recovered the growth-inhibitory effects of TGF-β in these cells [37]. Our studies of Smad4 protein expression in our *in vitro* model of HPV16-mediated transformation indicate that immortalization by HPV16 DNA triggers an early increase in Smad4 protein in HKc/HPV16, as compared to normal HKc, and this increase is maintained at later stages of *in vitro* progression. The functional significance of this finding remains to be determined.

Smad7 works as a feedback loop attenuating TGF-β signaling. Both deletions and amplifications of the Smad7 gene have been reported in colorectal tumors [14]. Nonetheless, amplifications are more common than deletions, as compared to the same genetic alterations of Smad2 and Smad4, which suggests that retention and even amplification of Smad7 is the selected event during colorectal tumorigenesis [14]. These findings are in agreement with frequent Smad7 overexpression found in endometrial and thyroid follicular carcinomas [14]. Studies have also found upregulation of Smad7 mRNA in pancreatic cancer as compared to normal tissue [38]. Furthermore, transfection of Smad7 into the TGF-β sensitive pancreatic cell line COLO-357 rendered them refractory to the antiproliferative effect of the cytokine, and drastically enhanced soft agar colony formation [38]. In another study, primary mouse keratinocytes were transduced with the Smad7 gene resulting in enhanced keratinocyte proliferation, blocked normal differentiation, and induced keratin 8, a marker of malignant conversion, but did not result in tumor formation [39]. When Smad7 was transduced together with HRAS, keratinocytes rapidly progressed to squamous cell carcinomas *in vivo*, whereas transduction with HRAS together with Smad6 or an empty vector control resulted in benign papillomas [39]. These findings demonstrate that Smad7 overexpression can accelerate tumor progression and cause malignant conversion in the context of other oncogenes [39]. Although no alterations in the Smad7 gene have been described in cervical cancer, we investigated if increased Smad7 levels could play a role in the progressive loss of growth inhibitory response to TGF-β1 that we observed as HKc/HPV16 progress to the HKc/DR stage. We found similar levels of Smad7 protein in HKc/HPV16 and HKc/GFI compared to normal HKc, with levels of Smad7 protein decreasing slightly in HKc/DR. Thus, our data do not support a role for Smad7 overexpression in TGF-β1 resistance in HKc/DR.

Many studies have demonstrated that activated TGFBR1 phosphorylates Smad2 and Smad3 resulting in formation of Smad4-containing heteromeric complexes that are translocated to the nucleus, where they drive transcriptional responses [11,40]. TGF-β treatment of untransfected Mv1Lu mink lung epithelial cells resulted in phosphorylation, nuclear shuttling and nuclear accumulation of Smad2 and Smad3 [11]. In addition, Smad4 also accumulated into the nucleus paralleling Smad2 and Smad3 shuttling [11]. Similarly, the spontaneously-immortalized TGF-β-responsive human keratinocyte HaCaT cell line accumulates Smad2/3 and Smad4 in the nucleus after treatment with TGF-β [41]. The peak of Smad2/3 nuclear accumulation and Smad2 phosphorylation takes place as early as 30 min following TGF-β treatment [41]. Additionally, experiments have demon-strated that TGF-β-treated cell lines expressing higher levels of TGFBR1 maintained nuclear accumulation of Smad2, Smad3 and Smad4 proteins, as well as Smad2 phosphorylation, for up to 6 h [42]. In contrast, nuclear accumulation of these Smads and phosphorylation of Smad2 could be maintained for only 1 or 2 h in other cell lines, which could be explained, at least in part, by the low expression of TGFBR1 in these cells [42].

Previous experiments in our laboratory found that a progressive loss of sensitivity to the growth inhibitory effects of TGF-β1, as HKc/HPV16 progress to the HKc/DR stage, strongly correlates with decreased expression of TGFBR1 messenger RNA and protein [24]. In order to further explore alterations in TGF-β signaling in our model system, we studied the kinetics of Smad3 and Smad4 nuclear accumulation, as well as the levels of Smad2 phosphorylation following TGF-β1 treatment. We observed a delay in Smad3 nuclear accumulation in HKc/DR as compared to normal HKc and HKc/HPV16; maximal Smad2 phosphorylation was also delayed in HKc/DR. Furthermore, the level of Smad2 phosphorylation after 6 h of TGF-β1 treatment was decreased in all four HKc/DR lines we studied, as compared to their HKc/HPV16 counterparts and to normal HKc. In contrast, nuclear accumulation of Smad4 was not delayed. These results indicate that the Smad system is mostly intact in HKc/DR: it is likely that the alterations we observe in Smad2 phosphorylation are a direct consequence of the loss of TGFBR1. To further assess the status of Smad signaling in HKc/DR, we compared the TGF-β1-induced activity of a luciferase reporter construct under the transcriptional control of six in-tandem SBEs in HKc/HPV16 and their corresponding HKc/DR [43]. These experiments showed that Smad-mediated TGF-β1 transcriptional activation is reduced by about 50% in HKc/DR, as compared to HKc/HPV16.

## Conclusions

In summary, our findings demonstrate that although HKc/DR are completely resistant to the growth inhibitory effects of TGF-β1 [24], the Smad pathway remains relatively intact in HKc/DR, including Smad translocation to the nucleus following TGF-β1 treatment, and partial induction of a luciferase reporter construct driven by 6SBEs. We will continue to utilize our *in vitro* model system for HPV16-mediated transformation and progression, which shares many gene expression changes with those found in premalignant cervical lesions and cervical cancer [44] to explore why HKc/DR are no longer responsive to the growth inhibitory effects of TGF-β1, even though substantial Smad signaling remains.

## Abbreviations

HPV16: Human papillomavirus type 16; TGF-β: Transforming growth factor-beta; TGF-β1: Transforming growth factor-beta 1; HKc: Human keratinocytes; HKc/HPV16: Human papillomavirus type 16-immortalized human keratinocytes; HKc/GFI: Growth factor independent human papillomavirus type 16-immortalized human keratinocytes; HKc/DR: Differentiation resistant human papillomavirus type 16-immortalized human keratinocytes; TGFBR1: TGF-β receptor type I; TGFBR2: TGF-β receptor type II; FBS: Fetal bovine serum; CM: Complete medium; EGF: Epidermal growth factor; BPE: Bovine pituitary extract; GFDM: Growth factor depleted medium; PBS: Phosphate-buffered saline; PVDF: Polyvinylidene fluoride; BSA: Bovine serum albumin; AF488: Alexa fluor 488; Cy3: Cyanine 3; T-ALL: T-cell acute lymphoblastic leukemia; SBEs: Smad-binding elements.

## Competing interests

The authors declare that they have no competing interests.

## Authors’ contributions

DA carried out the Western blot analysis for Smads, the immunofluorescence and confocal microscopy studies of Smad trafficking, and drafted the manuscript. RV performed the luciferase reporter assays. LP and KEC conceived of the study, participated in its design and coordination, and finalized the manuscript. All authors read and approved the final manuscript.

## Pre-publication history

The pre-publication history for this paper can be accessed here:

http://www.biomedcentral.com/1471-2407/13/424/prepub
